# Oral Hygiene and Dietary Behaviors Among Romanian Schoolchildren: A Cross-Sectional Study

**DOI:** 10.3390/children12121712

**Published:** 2025-12-18

**Authors:** Ioana Elena Lile, Carolina Cojocariu, Diana Marian, Tiberiu Hosszu, Ademir Horia Stana, Otilia Stana

**Affiliations:** 1 Department of Dental Medicine, Faculty of Dentistry, “Vasile Goldis” Western University of Arad, 310025 Arad, Romania; lile.ioana@uvvg.ro (I.E.L.); cojocariu.carolina@uvvg.ro (C.C.); stana.otilia@uvvg.ro (O.S.); 2 Department of Medicine, Faculty of Medicine, “Vasile Goldiș” Western University of Arad, 94-96 Revolutiei Blvd., 310025 Arad, Romania; stana.ademir@uvvg.ro

**Keywords:** oral health, dental caries, gingival inflammation, plaque index, oral hygiene behavior, schoolchildren, preventive dentistry

## Abstract

**Highlights:**

**What are the main findings?**

• Over two-thirds (69.8%) of Romanian schoolchildren presented visible plaque and gingival inflammation, despite a high reported frequency of toothbrushing.
• The youngest age group (5–7 years) exhibited the highest prevalence of active carious lesions, revealing a critical need for early preventive action.

**What are the implications of the main findings?**

• Oral health programs should focus on improving brushing techniques and promoting the regular use of interdental hygiene aids through school-based education.
• Early prevention strategies and parental engagement are essential to reduce caries incidence and establish sustainable oral hygiene behaviors from early childhood.

**Abstract:**

**Background/Objectives:** Oral health in children remains a key public health concern, particularly in regions with limited access to preventive programs. Despite improvements in dental care availability, the prevalence of plaque accumulation, gingival inflammation, and carious lesions remains high. This study provides updated regional data for Western Romania—a population previously underrepresented in oral health surveillance—and aims to evaluate oral hygiene behaviors, dietary habits, and clinical oral health indicators among Romanian schoolchildren, identifying potential areas for preventive action. **Methods:** An observational cross-sectional study was conducted in October 2025 on 202 schoolchildren aged 5–14 years from Western Romania. Data were collected through a structured questionnaire assessing socio-demographic characteristics, oral hygiene practices, and dietary behaviors, followed by a standardized intraoral examination. Plaque Index (PI) and Gingival Index (GI) were recorded, and statistical analysis was performed using chi-square tests (*p* < 0.05). **Results:** Most participants (83.7%) reported brushing their teeth at least twice daily, whereas only 24.8% used dental floss and 13.4% used interdental aids. The prevalence of carious lesions or restorations was 66.8%, visible plaque was 69.8%, and gingival inflammation was 50.0%. A significant positive correlation was observed between PI and GI (r = 0.58, *p* < 0.001). Children aged 5–7 years exhibited the highest rate of active carious lesions (71.2%, *p* = 0.014). **Conclusions:** Although brushing frequency among Romanian schoolchildren was generally satisfactory, inadequate interdental hygiene and suboptimal plaque control were common. School-based preventive programs emphasizing proper brushing technique, dietary counseling, and early education may contribute to improved oral health outcomes in this population.

## 1. Introduction

Oral health represents a fundamental component of general health and overall quality of life, influencing essential aspects such as mastication, speech, nutrition, social communication, and self-esteem [1,2]. Poor oral health in childhood may have long-term implications, potentially affecting growth, cognitive development, and school performance [3]. Despite remarkable progress in dental materials, preventive protocols, and community-based oral health education, dental caries and periodontal inflammation remain among the most prevalent chronic conditions worldwide, particularly in pediatric populations [4,5]. According to the Global Burden of Disease Study, untreated dental caries in primary teeth affect nearly half of all children globally, reflecting a major public health concern [6].

The World Health Organization (WHO) and the FDI World Dental Federation have emphasized the need for comprehensive oral health strategies that address behavioral, environmental, and socioeconomic determinants of disease, beyond its biological expression [7,8]. These strategies are especially relevant for developing and transitional countries, where disparities in preventive care and oral health education persist. In Romania, as in many Eastern European nations, limited access to regular dental check-ups, low oral health literacy, and economic inequalities are associated with early onset of carious lesions and challenges in maintaining adequate plaque control among children [9,10,11].

School-based oral health promotion programs, such as supervised toothbrushing, fluoride varnish application, and nutritional education, have demonstrated significant improvements in oral hygiene indicators when properly implemented [12,13]. However, their success largely depends on understanding the baseline knowledge, attitudes, and practices (KAPs) of children, parents, and educators regarding oral hygiene routines and diet [14]. Studies across Europe reveal that while children often report brushing twice daily, incorrect techniques, insufficient duration, and limited interdental cleaning may diminish the benefits of these behaviors [15,16]. Parental awareness and involvement also play a crucial role, as children’s hygiene practices frequently mirror those of their caregivers [17].

Dietary behaviors represent another key determinant of oral health. Frequent consumption of sugar-rich snacks and acidic beverages contributes to dental caries and early gingival inflammation [18,19]. The frequency of sugar exposure, rather than the total amount consumed, is considered a critical factor in caries risk [20]. Such dietary patterns are often influenced by socioeconomic status, educational background, and the urban–rural environments [21,22]. Studies from Eastern Europe indicate that children from rural or low-income families may have reduced access to preventive dental visits and higher consumption of cariogenic diets [23,24].

While the biological foundation of dental caries, namely, the demineralization of hard dental tissues through acid production by bacterial biofilm, is well established, the interplay between biological, behavioral, and environmental factors remains complex [25,26]. Recent evidence suggests that modifiable lifestyle determinants, such as brushing frequency, fluoride exposure, and dietary regulation, may have a substantial influence on caries incidence, although their relative contribution varies across populations with differing access to preventive resources [27,28].

In this context, the collection of national and regional data is essential for shaping evidence-based, population-specific preventive programs. Romania lacks consistent epidemiological surveillance of children’s oral health, and comparable data from Central and Eastern Europe are also scarce. Evaluating the current status of children’s oral hygiene behaviors and dietary habits is, therefore, important for designing effective public health interventions and integrating preventive education into school curricula.

The aim of this observational cross-sectional study was to examine associations between oral hygiene behaviors, dietary habits, and clinical oral health indicators among Romanian schoolchildren. By correlating questionnaire responses with clinical findings—including plaque and gingival indices—this research sought to describe risk profiles and the modifiable determinants associated with oral disease.

Research hypothesis: Children with poorer interdental hygiene and more frequent sugar intake present higher plaque and gingival index scores. This hypothesis refers strictly to expected statistical associations rather than causal relationships.

Research question: Which modifiable oral hygiene and dietary factors are most strongly correlated with clinical indicators of oral health among Romanian schoolchildren?

## 2. Materials and Methods

### 2.1. Study Design and Ethical Approval

This observational cross-sectional study was conducted in accordance with the ethical principles of the Declaration of Helsinki (2013 revision). The research protocol was approved by the Scientific Research Ethics Committee of “Vasile Goldiș” Western University of Arad (Approval No. 17, issued on 2 October 2025). All participants were recruited voluntarily after their parents or legal guardians received a detailed explanation of the study objectives and procedures and provided written informed consent prior to data collection.

A total of 202 schoolchildren aged 5–14 years were enrolled from both urban and rural schools across Western Romania. To ensure diversity, participants were selected through stratified random sampling based on geographic area and socioeconomic status. All children who met the eligibility criteria in the selected classes were invited to participate, resulting in a 97% acceptance rate. No participants were excluded after enrollment, and no withdrawals occurred during data collection.

The minimum sample size was estimated using a 95% confidence level, a 5% margin of error, and an assumed 50% caries prevalence, resulting in a required minimum of 196 participants. The final sample (*n* = 202) met this requirement.

Inclusion criteria:

(1) Children aged 5–14 years;
(2) Attendance at regular educational institutions;
(3) Presence of at least 20 erupted teeth suitable for clinical evaluation, including mixed dentition.

Exclusion criteria:

(a) Ongoing orthodontic treatment;
(b) Antibiotic use in the last 3 months;
(c) Systemic or neurological disorders affecting salivary flow or oral motor function (e.g., xerostomia, cerebral palsy, autism spectrum disorder);
(d) Absence of parental consent.

The study was conducted in October 2025 under the supervision of trained dental specialists and academic staff from the Faculty of Dental Medicine, “Vasile Goldiș” Western University of Arad. Confidentiality and anonymity were maintained throughout the research process.

### 2.2. Data Collection

Data collection followed a standardized protocol developed by the research team to ensure consistency across all participating schools. A structured, pre-tested questionnaire was administered in classrooms under the supervision of teachers and research staff. For children under 10 years of age, the questionnaire was completed by parents or guardians; older participants completed it independently, with teacher assistance when needed. Each questionnaire and clinical evaluation form was assigned a unique identification code to ensure participant confidentiality.

The questionnaire was adapted from Oral health surveys: basic methods-5th edition (2013) and culturally adjusted for use in Romanian populations. It comprised 31 closed-ended questions grouped into four sections:

• Demographic and socioeconomic data (age, sex, residence, parental education, and occupational status);
• Oral hygiene practices (frequency, duration, and technique of toothbrushing; type of toothbrush; use of dental floss, mouth rinses, and interdental brushes);
• Dietary behaviors (number of meals per day, snack frequency, sugar intake, and beverage choices);
• Self-reported oral health indicators (presence of bleeding gums, dental sensitivity, gingival swelling, and history of dental visits).

The questionnaire underwent translation and back-translation by bilingual experts to ensure linguistic and conceptual equivalence. A pilot test was conducted on a sample of 20 schoolchildren to evaluate clarity and cultural appropriateness, after which minor wording adjustments were made.

Teachers supervised questionnaire completion to minimize omissions. All questionnaire forms were verified by the research team at the time of collection, and clinical examination sheets were checked for completeness immediately after assessment to minimize missing information. The final dataset contained no missing values for primary clinical outcomes (Plaque Index, Gingival Index, DMFT/dmft). In children with mixed dentition, caries experience was recorded separately for primary (dmft) and permanent teeth (DMFT). No combined scores were calculated. A small number of missing responses (<1%) occurred for non-essential questionnaire items, mainly related to self-reported dietary habits. These missing entries were treated as missing completely at random and analyzed accordingly. Responses were encoded into a centralized database for analysis.

### 2.3. Clinical Examination

A standardized intraoral clinical examination was performed on all participants under natural light by two calibrated pediatric dentists. Examinations followed the World Health Organization (WHO) Oral Health Survey criteria, using sterile dental mirrors and WHO CPI probes (ball-end, 0.5 mm). The following parameters were recorded:

• Plaque Index (PI) according to Silness and Löe (1964); because the clinical examination in this study was performed under school conditions, without air-drying and using only a probe for detection, both PI = 1 and PI ≥ 2 were considered clinically relevant.
• Gingival Index (GI) according to Löe and Silness (1963).
• Dental caries status evaluated using the Decayed, Missing, and Filled Teeth indices for permanent (DMFT) and primary (dmft) dentition;
• Additional clinical signs include gingival bleeding, enamel hypomineralization, and dentin hypersensitivity.

Examiner calibration was assessed in a pilot group of 20 children (ages 7–10). Inter-examiner reliability (Cohen’s kappa) reached 0.87 for caries detection and 0.84 for gingival inflammation, indicating excellent agreement. All clinical examinations were performed in classrooms under hygienic conditions using sterile gloves, masks, and single-use instruments.

### 2.4. Data Management and Statistical Analysis

Data were analyzed using IBM SPSS Statistics version 29. Descriptive statistics (mean, standard deviation, frequency, and percentage) were calculated for all variables. Group differences were assessed using χ^2^ tests for categorical outcomes and independent-samples t-tests for continuous variables (DMFT and dmft). PI and GI, as ordinal indices, were analyzed using χ^2^ tests. Pearson correlation was used to evaluate the relationship between mean PI and mean GI after verifying linearity and normality of the variables. A significance level of *p* < 0.05 was applied. Pearson correlation coefficients were calculated to assess the relationship between the Plaque Index (PI) and Gingival Index (GI). Normality of the data distribution was assessed using the Shapiro–Wilk test. All reporting and statistical processes adhered to the STROBE standard for cross-sectional observational studies (Supplementary Material S1).

## 3. Results

### 3.1. Participant Characteristics

A total of 202 schoolchildren were included in the analysis. The mean age was 8.37 ± 2.14 years (range: 5–14 years). The sample included 52.5% girls and 47.5% boys, with the majority residing in urban areas (76.7%) and enrolled in primary school (90.1%).

Table 1 summarizes the demographic distribution of the study population.

### 3.2. Oral Hygiene Practices

#### 3.2.1. Frequency and Duration of Brushing

Most participants (83.7%) reported brushing their teeth at least twice daily, while 16.3% brushed once a day or less. The majority (60.9%) brushed for around two minutes, and 24.3% used an electric toothbrush (Table 2).

#### 3.2.2. Brushing Technique and Use of Auxiliary Hygiene Aids

The Fones method was the most frequently used brushing technique (36.6%), followed by the Bass technique (31.7%), while the remainder employed horizontal or mixed brushing motions (Table 2).

**Figure 1:**
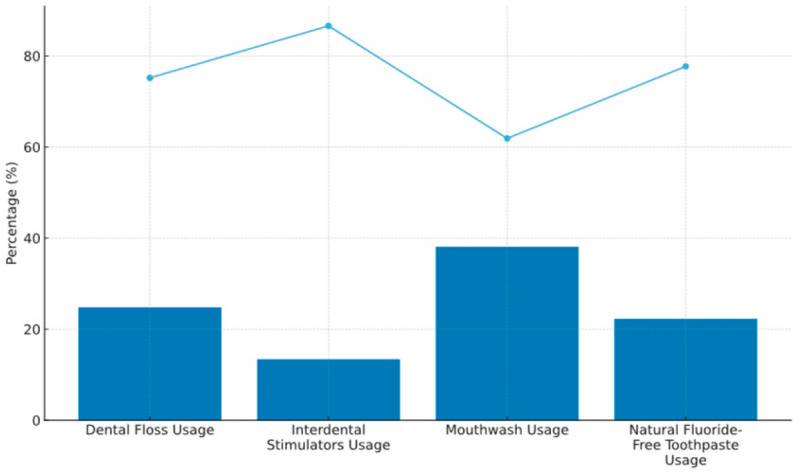
Usage of auxiliary oral hygiene aids among the study participants (*n* = 202), including dental floss, interdental stimulators, mouthwash, and fluoridated toothpaste {rectangular intensity: blue—YES, light blue line—NO}. Values are expressed as percentages of the total sample.

Auxiliary oral hygiene aids were less commonly used:

• Dental floss: 24.8%
• Interdental brushes/stimulators: 13.4%
• Mouth rinsing: 38.1% (Figure 1).

Urban participants reported higher use of auxiliary aids compared with rural participants (*p* < 0.05).

**Table 2:** Oral hygiene habits and behaviors.

Parameter	Category	*n* (%)
Brushing frequency	≥2 times/day	169 (83.7)
	≤1 time/day	33 (16.3)
Brushing duration	2 min	123 (60.9)
	<2 min	79 (39.1)
Type of toothbrush	Manual	153 (75.7)
	Electric	49 (24.3)
Brushing technique	Fones	74 (36.6)
	Bass	64 (31.7)
	Other	64 (31.7)
Dental floss use	Yes	50 (24.8)
Interdental aids	Yes	27 (13.4)
Mouth rinse use	Yes	77 (38.1)

**Figure 2:**
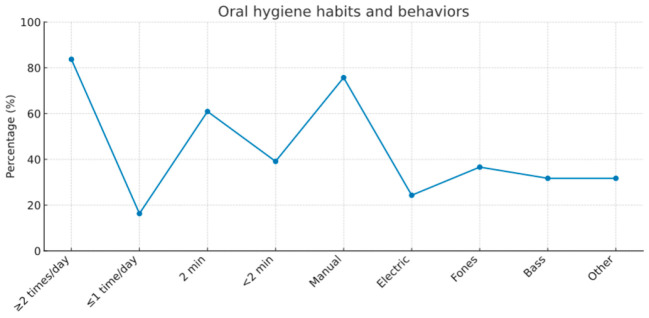
Percent distribution of oral hygiene behaviors, including brushing frequency, duration, toothbrush type, and brushing technique among schoolchildren (*n* = 202).

As shown in Figure 2, the percent distribution of oral hygiene behaviors- including brushing frequency, duration, toothbrush type, and brushing technique -among schoolchildren (*n *= 202) is illustrated.

### 3.3. Dietary Habits

**Figure 3:**
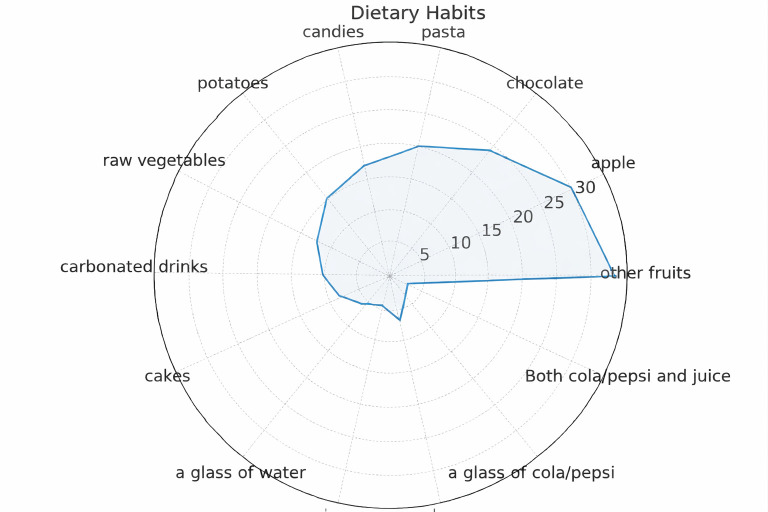
Dietary behaviors of the participants (*n* = 202), including frequency of fruit and pastry/sweet consumption and the proportion of children reporting drinking water after meals. Values expressed as percentages.

Regarding dietary behavior, 32.6% of participants reported frequent consumption of fresh fruits, while 26.3% regularly consumed pastries and sweets. More than half (53%) reported drinking water after meals (Figure 3).

Children who consumed sugary snacks more frequently had a higher prevalence of visible plaque (*p* = 0.021), suggesting an association between sugar exposure and plaque accumulation.

### 3.4. Clinical Findings

Clinical examinations revealed that 66.8% of participants had carious lesions or restorations, 69.8% exhibited visible dental plaque, and 50.0% presented gingival inflammation. Additionally, 26.7% reported bleeding during brushing, and 41.6% experienced dentin hypersensitivity (Table 3).

The Plaque Index (PI) and Gingival Index (GI) demonstrated a moderate positive correlation (r = 0.58, *p* < 0.001). The youngest age group (5–7 years) showed the highest prevalence of active carious lesions (71.2%, *p* = 0.014 vs. older groups).

**Table 3:** Clinical parameters of oral health.

Parameter	Affected *n* (%)	*p*-Value (Group Comparison)
Carious lesions/restorations	135 (66.8)	—
Visible plaque (PI ≥ 1)	141 (69.8)	<0.001
Gingival inflammation (GI ≥ 1)	101 (50.0)	<0.001
Bleeding on brushing	54 (26.7)	0.043
Dentin hypersensitivity	84 (41.6)	0.058

### 3.5. Summary of Key Findings

• Brushing frequency was generally satisfactory, but the use of interdental hygiene aids was limited.
• Frequent consumption of sugary snacks was associated with higher visible plaque levels.
• The high prevalence of plaque and gingival inflammation suggests suboptimal plaque control despite reported brushing frequency.
• Children aged 5–7 years showed the highest caries burden.
• Findings support the need for school-based preventive programs emphasizing brushing technique, interdental cleaning, and dietary moderation.

## 4. Discussion

This observational cross-sectional study among Romanian schoolchildren (*n* = 202; 5–14 years) identified a persistent discrepancy between self-reported oral hygiene behaviors and clinical oral health outcomes. Although most participants reported brushing at least twice daily (83.7%), visible plaque (69.8%) and gingival inflammation (50.0%) remained highly prevalent, while carious lesions or restorations affected two-thirds of the sample (66.8%). Limited use of interdental cleaning aids (floss 24.8%; interdental brushes 13.4%) and the significant correlation between the Plaque Index and Gingival Index (r = 0.58, *p* < 0.001) highlight the importance of comprehensive plaque control. The youngest age group (5–7 years) presented the highest prevalence of active carious lesions (71.2%), suggesting that early preventive initiatives may be particularly relevant.

Our findings are consistent with international research indicating that self-reported brushing frequency alone may not be a reliable indicator of oral health status when technique, duration, and interdental cleaning are inadequate [1,2]. The association between PI and GI aligns with the classical relationship described by Löe and Silness, whereby higher plaque levels are commonly accompanied by increased gingival inflammation [4]. Similar age-related gradients in caries prevalence have been reported in European populations, where early-onset disease in primary dentition persists due to insufficient structured prevention and limited parental supervision [8,9,10,11]. The relatively high proportion of children who reported drinking water after meals (>50%) is encouraging and corresponds with behaviors previously associated with improved oral pH recovery [18].

Although urban children reported higher use of auxiliary hygiene aids than their rural peers, clinical outcomes remained broadly comparable, suggesting that access to hygiene tools does not necessarily translate into consistently effective daily practice. Comparative studies from Poland, Hungary, and Serbia confirm similar patterns—higher caries prevalence and poorer hygiene indicators among rural children, often linked to lower parental education and limited preventive services [29,30,31]. These regional parallels reinforce the need for multilevel prevention strategies addressing both behavioral and socioeconomic disparities.

Parental modeling plays a pivotal role in shaping children’s daily hygiene routines and dental attendance. The limited adoption of interdental aids in our cohort was likely associated with persistent interdental biofilm and gingival bleeding despite apparently adequate brushing frequency. Strengthening parental oral health literacy—particularly among mothers—could enhance the sustainability of school-based interventions. Simple, structured tools such as take-home checklists, reminder cards, or teacher-facilitated reinforcement activities may help bridge the gap between awareness and consistent daily practice.

Dietary behaviors remain a major determinant of oral health. Frequent sugary snack consumption was associated with a higher prevalence of plaque (*p* = 0.021), consistent with established associations between sugar exposure frequency and caries risk [18,19,20]. Studies from Central and Eastern Europe report similar patterns, often linked to socioeconomic and educational disparities [29,30,31,32]. Integrating dietary counseling with hygiene education—through small, practical steps such as promoting water after meals or encouraging fruit and dairy snacks—may support more sustainable health behavior change.

Taken together, the present data highlight the relevance of early oral-health education within a skills-focused preventive framework, particularly in school settings. Within such a framework, several domains may be considered when designing age-appropriate programs:

(1) Hands-on brushing instruction using simple, age-appropriate methods;
(2) Progressive familiarization with interdental hygiene tools;
(3) Brief, practical dietary-awareness activities;
(4) Parental engagement through accessible educational materials.

As younger age groups exhibited the highest caries burden, early adoption and periodic reinforcement of such behaviors may support better outcomes. Implementing simple monitoring tools—such as periodic Plaque Index screenings or classroom adherence logs—may help maintain program consistency and identify groups requiring additional support.

Study limitations.

This study presents several methodological limitations that should be acknowledged. First, its cross-sectional design does not allow for causal inference between oral hygiene behaviors, dietary patterns, socioeconomic characteristics, and clinical oral health outcomes; all results should be interpreted as associations. Second, the sample included a higher proportion of urban participants (76.7%), which may reduce the representativeness of oral health status for the entire Western Romanian region. Third, behavioral information was obtained through self-reported questionnaires, which are susceptible to recall bias and social desirability bias, particularly among younger children whose responses relied on parental reporting.

Clinical examinations were performed under school conditions without radiographic assessment, possibly underestimating early carious lesions. Fluoride exposure, sealant application, and access to dental services were not assessed and should be considered in future studies.

Future research should include larger, multi-regional cohorts and longitudinal designs to evaluate behavioral sustainability and intervention efficacy over time. Randomized controlled trials comparing supervised brushing intensity, interdental hygiene adoption, and combined hygiene–diet interventions could clarify the optimal strategies for school-based prevention in Eastern Europe.

In summary, the findings demonstrate that awareness without effective practice leaves a substantial preventable disease burden. Emphasizing technique quality, interdental hygiene, and reduced sugar frequency—delivered collaboratively through school and family partnerships—offers a pragmatic, evidence-based pathway to improved oral health among Romanian children.

## 5. Conclusions

This observational cross-sectional study provides an updated overview of oral health status, hygiene behaviors, and dietary patterns among Romanian schoolchildren aged 5–14 years. Although most children reported brushing at least twice daily, substantial levels of plaque accumulation, gingival inflammation, and carious lesions were observed, indicating that preventive behaviors may not be applied effectively or consistently. Interdental cleaning practices remained limited, daily sugar intake was frequent, and socioeconomic disparities—particularly in maternal education—continued to influence children’s oral health outcomes. The positive association between the Plaque Index and Gingival Index underscores the central role of plaque control in maintaining gingival health.

To help reduce disease burden and behavioral inequalities, a two-tiered preventive approach may be considered. First, universal school-based interventions could include daily supervised brushing with fluoridated toothpaste (1000–1450 ppm), sugar-reduction education, and regular oral health workshops conducted collaboratively by dental professionals and teachers.

Second, targeted programs for high-risk groups—especially children from rural or socioeconomically disadvantaged communities—should focus on those presenting high amounts of plaque or caries indices. Strengthening parental engagement may support improved oral hygiene habits, although this recommendation extends beyond the scope of the present cross-sectional findings.

Overall, adopting integrated preventive strategies that combine education, parental participation, and structured school-based monitoring aligns with international public health priorities and may support more equitable oral health outcomes for children.

## Figures and Tables

**Table 1:** Demographic characteristics of the study population.

Variable	Category	n (%)
Age (years)	5–7	86 (42.6)
	8–10	73 (36.1)
	11–14	43 (21.3)
Gender	Male	96 (47.5)
	Female	106 (52.5)
Residence	Urban	155 (76.7)
	Rural	47 (23.3)
School type	Primary school	182 (90.1)
	Secondary school	20 (9.9)
Parental education	High school University degree	359 (88.9) 45 (11.1)
Parental occupation	Employed Unemployed Part-time	382 (94.6) 5 (1.2) 17(4.2)

## Data Availability

The original contributions presented in this study are included in the article. Further inquiries can be directed to the corresponding author.

## Supplementary Materials

The following supporting information can be downloaded at: https://www.mdpi.com/article/10.3390/children12121712/s1, Supplementary Material S1. STROBE Checklist.

## Abbreviations

The following abbreviations are used in this manuscript:

CPI	Community Periodontal Index
DMFT	Decayed, Missing, and Filled Teeth (permanent dentition)
dmft	Decayed, Missing, and Filled Teeth (primary dentition)
FDI	FDI World Dental Federation
GI	Gingival Index
KAP	Knowledge, Attitudes, and Practices
PI	Plaque Index
SD	Standard Deviation
WHO	World Health Organization
