# Supplementary material for: Oral Hygiene and Dietary Behaviors Among Romanian Schoolchildren: A Cross-Sectional Study
Source: Children (Basel). 2025 Dec 18;12(12):1712. doi: 10.3390/children12121712 (PMC12731438; doi:10.3390/children12121712)
Supplement: Supplementary file 1 [file children-12-01712-s001.zip › children-3990094-supplementary.pdf]

STROBE Checklist – Cross-sectional Study

Oral Hygiene and Dietary

Behaviors among Romanian Schoolchildren: A Cross-Sectional Study

Item No	Recommendation	Where Reported in Manuscript
1a	Indicate study design in title/abstract	Abstract (cross-sectional descriptive study)
1b	Provide informative summary	Abstract
2	Scientific background and rationale	Introduction, pp. 2–3
3	State specific objectives and hypotheses	Introduction, end section
4	Present key elements of study design	Section 2.1
5	Describe setting, locations, dates	Section 2.1
6a	Eligibility criteria; sources/methods of selection	Section 2.1
7	Define outcomes, predictors, confounders	Sections 2.2–2.3
8	Data sources and measurement details	Sections 2.2–2.3
9	Describe efforts to address bias	Methods + Study Limitations
10	Explain how study size was determined	Section 2.1

11	Explain handling of quantitative variables	Section 2.4
12a	Describe statistical methods	Section 2.4
12b	Describe subgroup/interactions analysis	Results + Discussion
12c	Explain missing data treatment	Section 2.2
13a	Report participant numbers at each stage	Section 2.1 + Results
13b	Reasons for non-participation	Section 2.1
14a	Descriptive participant characteristics	Results, Table 1
14b	Number of participants with missing data	Section 2.2
15	Outcome data	Results, Table 2,3
16	Main results	Results
17	Other analyses	Results + Discussion
18	Key results summary	Discussion, opening paragraph
19	Study limitations	Discussion, Limitations section
20	Interpretation of results	Discussion
21	Generalisability	Discussion
22	Funding	Funding section at end of manuscript
